# Pneumatic vs laser lithotripsy for mid-ureteric stones: Clinical and cost effectiveness results of a prospective trial in a developing country

**DOI:** 10.1080/2090598X.2020.1749800

**Published:** 2020-04-15

**Authors:** Hani H. Nour, Ahmed I. Kamel, Hazem Elmansy, Mohamad H. Badawy, Waleed Shabana, Ayman Abdelwahab, Ahmed Elbaz, Tarek Eleithy, Mamdouh Rushdy

**Affiliations:** a Department of Urology, Theodor Bilharz Research Institute, Giza, Egypt; b Department of Urology, Thundar Bay Regional Health Sciences Center, Thunder Bay, ON, Canada; c Department of Urology, Zagazig University, Zagazig, Egypt

**Keywords:** Ureter, stone, laser, pneumatic, costs

## Abstract

**Objective:**

To compare the management of large ureteric stones (>10 mm) with ureterorenoscopy (URS) and laser or pneumatic lithotripsy, and their associated costs.

**Patients and methods:**

Our prospective study followed the tenets of the Declaration of Helsinki and included 101 patients with large mid-ureteric stones eligible for URS and lithotripsy, and was conducted between January 2018 and August 2019. Patients were randomly divided into two groups: Group 1 had laser lithotripsy, while the Group 2 had lithotripsy using a pneumatic energy source.

**Results:**

Operative time was significantly longer in cases using pneumatic lithotripsy (*P* < 0.001). The stone-free rate (SFR) on the first postoperative day was 94% and 92.5% for laser and pneumatic lithotripsy respectively, and there were no statistically significant differences in terms of early (day 1) or late (day 30) SFRs between the groups. Complications were classified according to the Clavien–Dindo Grading System, all complications were Grade <III, with no statistically significant difference between the groups (*P* = 0.742). The use of pneumatic lithotripsy had lower treatment costs. The number of auxiliary procedures required to reach a stone-free status was statistically equivalent in both groups.

**Conclusion:**

The type of lithotripsy did not affect the SFR or complications. However, laser lithotripsy was much more expensive than pneumatic lithotripsy.

**Abbreviations:**

KUB: plain abdominal radiograph of the kidneys, ureters and bladder; SFR: stone-free rate; SWL: shockwave lithotripsy; URS: Ureterorenoscopy; US: ultrasonography

## Introduction

The main objective of stone treatment is to achieve the highest stone-free rate (SFR) with minimal morbidity. In a market of highly sophisticated materials and equipment, the costs incurred to achieve a stone-free status must be taken into consideration. This is particularly true in developing countries, where healthcare systems are subsidised by governments. In the absence of a national healthcare insurance system, patients may be expected to contribute financially to their care [1].

Management of large ureteric stones (>10 mm) represents a treatment challenge for physicians. The selection of an appropriate treatment strategy depends upon several factors including stone size, stone composition, the presence of obstruction, as well as patient anatomy and surgeon experience. The availability of materials and financial factors also have to be considered [2,3].

Open and laparoscopic surgical removals are considered highly morbid in relation to minimally invasive procedures; yet, in cases with associated anatomical abnormalities or in presence of ureteric strictures, conventional surgery is a valid option. Shockwave lithotripsy (SWL) produces excellent results in terms of SFR for proximal ureteric stones, yet in mid and distal stones SWL is hindered by overlying viscera and underlying bony structures [3–5].

For large mid-ureteric stones (>10 mm), ureterorenoscopy (URS), either flexible and/or semi-rigid with lithotripsy, is the most widely accepted first-line treatment option.

In comparison to SWL, URS is associated with earlier SFR, less auxiliary procedures and lower morbidity when compared to conventional or laparoscopic surgeries [6].

Several types of energy sources are typically used during lithotripsy. Pneumatic lithotripsy provides high SFRs; however, there is a considerable incidence of stone migration. Since the introduction of laser lithotripsy, a shift towards its use has been seen, especially when flexible URS is used, and when medical conditions such as inability to stop anticoagulation therapy are present. Laser lithotripsy is associated with high SRFs, a lower incidence of stone migration, yet a considerable increase in costs and operative time has also been reported [7,8].

In Egypt, health services are subsidised by the government and are almost free-of-charge at university and public hospitals. The country’s limited resources, large population, in addition to the increased costs of supplies and materials, present a burden on Egypt’s developing economy [9]. To validate clear indications for the use of costly high-technological materials, our present study evaluated the results of both pneumatic and holmium laser lithotripsy at a tertiary university centre in Egypt in terms of SFRs, auxiliary procedures and costs.

## Patients and methods

Our prospective study followed the tenets of the Declaration of Helsinki and included 101 patients with large mid-ureteric stone eligible for URS and lithotripsy, and was conducted between January 2018 and August 2019.

The inclusion criteria were: age >18 years, single mid-ureteric stone >10 mm on non-contrast CT of the kidney-ureter-bladder, or multiple stones with the sum of their largest diameter >10 mm, no history of open or laparoscopic stone surgery on the stone site.

Patients were randomly divided into two groups: Group 1 had laser lithotripsy, while Group 2 had lithotripsy using a pneumatic energy source. Randomisation was accomplished using a simple randomisation method (shuffled cards). Randomisation and allocation were concealed by an independent registered nurse. The outcome assessor was blinded to the intervention after allocation.

The sample size was calculated by comparing the SFR proportion between pneumatic and holmium laser lithotripsy based on prior research that studied the outcomes of treatment of ureteric stones with different fragmentation methods [10]. An effect size of 18.1% and 48.1% of SFR was postulated according to the aforementioned study. For the power of the test to be 80% at a CI of 95%, the calculated sample was 38 in each group. After allowing for a 35% drop-out rate, 53 patients were allocated to each group.

Laser lithotripsy was performed using the MultiPulse HoPLUS® (Asclepion Laser Technologies GmbH, Jena, Germany), a 110-W holmium:yttrium-aluminium-garnet (YAG)-pulsed laser machine, with 365 µm fibres. Both dusting and fragmentation techniques were used.

A generic and locally approved Egyptian lithotriptor was used for pneumatic lithotripsy. Operative time was calculated from the time of anaesthesia induction to securing the catheter to the patient’s leg. X-ray exposure time was calculated by the X-ray technician at the end of the procedure and registered in the patient’s file. Residuals fragments, if any, were removed using forceps and/or stone basket extractor.

All the supplies including disposable equipment used during the procedure were documented by the circulating nurse and reviewed by the surgeon. The list of supplies was then sent to the institution’s accounts department.

Complications were graded according to the Clavien–Dindo Grading System [11]. Intraoperative complications including stone migration, ureteric injuries (false passage, mucosal tear, extravasations, and ureteric avulsion) were documented by the surgeon. Postoperative complications were documented separately.

Residual fragments were defined as stones ≥4 mm present on plain abdominal radiograph of the kidneys, ureters and bladder (KUB) and ultrasonography (US) [12] on postoperative day 1. In the absence of any residual fragments, patients were discharged and seen at their 1-month follow-up visit. In the presence of residual fragments or stone migration, the need for an auxiliary procedure was determined by the surgeon and communicated to the patient. All patients underwent KUB and US at postoperative day 30; patients with stone fragments at 1 month were considered treatment failures.

On discharge, the Accounts Department issued a bill to the patients that listed all direct costs (materials, drugs, consumables, accommodation) and indirect costs (depreciation of the laser machine and pneumatic energy source). A copy of the bill was placed in each patient’s file.

In cases where auxiliary procedures were required (i.e. SWL or JJ-stent removal), the sum of the patient’s bills were calculated to determine the cost of stone-free status at 1-month postoperatively.

The cost of the procedure included all direct costs including materials, disposable supplies, and use of operating theatre, treatment of complications if present, whereas, depreciation of the laser machine and pneumatic energy source were included as indirect costs. All costs were recorded in Egyptian Pounds (EGP), and approximated to the nearest 5 EGP.

### Statistical analysis

Using the Student’s *t*-test (independent sample *t*-test) the quantitative (continuous) variables of both groups were compared. The results were tabulated as means ± standard deviations (SDs).

The qualitative (categorical) data of the two groups were compared using chi-square and Fisher’s exact tests (only when the expected was ≤5). The results obtained were then calculated as percentages.

Differences were considered significant at a *P* < 0.05 and the Statistical Package for the Social Sciences (SPSS®), version 14 was used (SPSS Inc., IBM Corp., Armonk, NY, USA).

## Results

**Figure 1.**
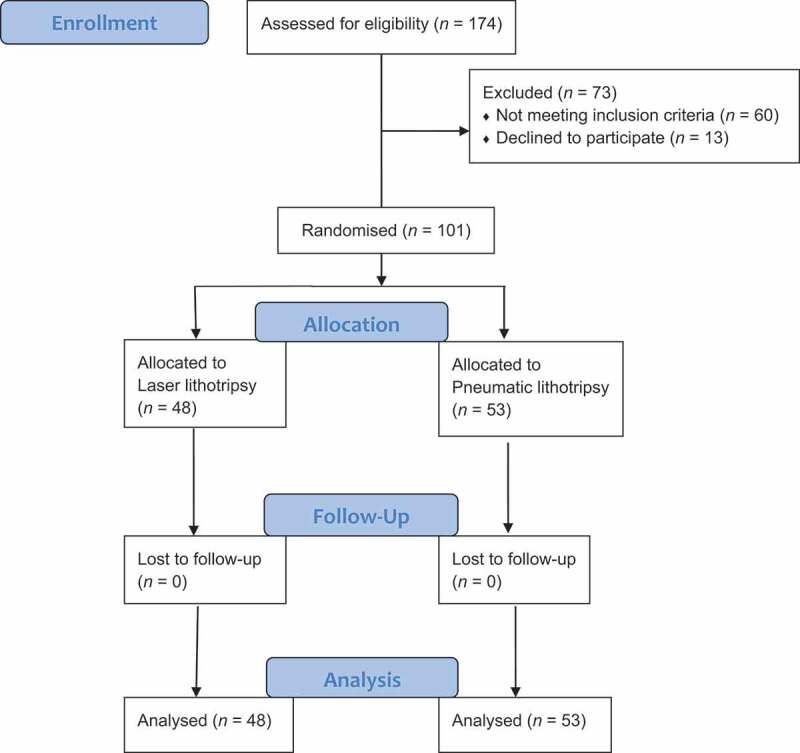
Consolidated standards of reporting trials (CONSORT) flow diagram.

In all, 101 patients (75 male and 26 female) with a median age of 39.1 years were included in the study (Figure 1). They presented on day 30 post-URS for follow-up. The demographic data of both arms is presented in Table 1, with no statistical difference between the groups.

**Table 1.** Patients’ characteristics.

Characteristic	Group 1 Laser lithotripsy (*N* = 48)	Group 2 Pneumatic lithotripsy (*N* = 53)	*P*
Age, years, mean (SD)	36.72 (13.31)	41.54 (11.22)	0.051
Sex, *n/N* (%) Male Female	35/48 (72.9)13/48 (27.1)	40/53 (75.5)13/53 (24.5)	0.769
Laterality, *n/N* (%) Right Left	18/48 (37.5)30/48 (62.5)	24/53 (45.3)29/53 (54.7)	0.428
Stone size, mm, mean (SD)	13.6 (2.43)	13.22 (2.81)	0.475
Number of stones, *n/N* (%) 1 2 3	38/48 (79.2)8/48 (16.7)2/48 (4.2)	37/53 (69.8)13/53 (24.5)3/53 (5.7)	0.56

An overall increased operative time was reported in Group 2 (pneumatic lithotripsy) compared to Group 1 (laser lithotripsy); including X-ray exposure time (Table 2).

**Table 2.** Operative results.

Variable	Group 1 Laser lithotripsy(*N* = 48)	Group 2 Pneumatic lithotripsy(*N* = 53)	*P*
Operative time, min, mean (SD)	43.75 (9.02)	55.18 (13.86)	<0.001*
X-ray exposure, min, mean (SD)	3.14 (1.0)	3.6 (1.18)	0.04
Irrigation volume, L, mean (SD)	12.52 (2.36)	12.94 (2.93)	0.431
Stenting, *n/N* (%)	40/48 (83.3)	46/53 (86.7)	0.625
Internal stent	15/40	14/46	0.524
External stent	25/40	32/46	0.386

*Value statistically significant at *P* < 0.05.

At the end of the procedure, a ureteric stent was inserted in 86 (85%) patients (*P* < 0.005). The decision of whether or not to insert a stent (internal or external) was based on the surgeon’s clinical evaluation of each patient. The type of energy source used during the procedure did not affect the surgeon’s decision nor the type of stenting selected (Table 2).

Stone migration to the upper ureter and kidney during the procedure occurred in four patients (two in each group), all had multiple stones >15 mm. An internal stent was inserted and these patients were re-evaluated on postoperative day 1.

At the end of the procedure, the ureter was inspected along its course, and retrograde pyelography was routinely performed. Mucosal breaches, false passages, the appearance of the periureteric fat, and contrast extravasation were documented and managed accordingly.

Complications according to the Clavien–Dindo Grading System occurred in 15 patients, seven in Group 1 and eight in Group 2. Intraoperative ureteric perforation occurred in two patients, who were treated with insertion of an internal stent, with one of them developing a fever postoperatively that was managed conservatively.

In all, 10 patients, five in each group, developed postoperative fever; all 10 patients already had an internal stent and were managed conservatively with an uneventful course. Two patients had persistent gross haematuria for >24 h postoperatively, which resolved with increased diuresis. All complications were Grade <III, with no statistical significance in terms of the number and type of complications between the groups (Table 3).

**Table 3.** Complications.

Variable	Group 1 Laser lithotripsy(*N* = 48)	Group 2 Pneumatic lithotripsy(*N* = 53)	*P*
Stone migration, *n/N* (%)	2/48 (4.2)	2/53 (3.7)	0.919
Fever, *n/N* (%)	5/48 (10.5)	5/53 (9.5)	0.869
Gross haematuria, *n/N* (%)	1/48 (2)	1/53 (1.9)	0.944
Ureteric injury, *n/N* (%)	1/48 (2)	2/53 (3.8)	0.617
Clavien–Dindo Grade <III, *n/N* (%)	7/48 (14.5)	8/53 (17)	0.742

On postoperative day 1, a combined KUB and US protocol was used to evaluate the presence of residual stones and calculate the SFR. A fragment of ≥4 mm was defined as a significant residual stone.

Using this protocol, the SFR was 94% in Group 1 and 92.5% in Group 2, with no statistical significance between the groups; the same was also found at 30-days postoperatively. In the presence of residual fragments and the absence of complications, it was the surgeon’s decision as to whether or not to perform an immediate auxiliary procedure or to re-evaluate the patients 2 weeks later.

The main auxiliary procedure performed was JJ-stent removal. One patient in Group 1 (laser lithotripsy) underwent extracorporeal SWL (ESWL) for a residual fragment in the kidney. A second-look URS and ESWL were performed in two patients for residual fragments, in the upper ureter and kidney, respectively, in Group 2 (pneumatic lithotripsy) (Table 4). Persistent residual fragments on imaging on postoperative day 30 were present in three patients (2.5%), one in Group 1 and two in Group 2 (*P* > 0.005).

**Table 4.** SFR and auxiliary procedures.

Variable	Group 1 Laser lithotripsy(*N* = 48)	Group 2 Pneumatic lithotripsy(*N* = 53)	*P*
Residual on day 1, *n/N* (%)	3/48 (6.3)	4/53 (7.5)	0.798
Auxiliary procedures, *n/N* (%)	15/48 (31.3)	14/53 (26.4)	0.592
Residual on day 30, *n/N* (%)	1/48 (2.1)	2/53 (3.8)	0.617
Auxiliary procedure type, *n/N* JJ-stent removal ESWL URS	15/151/150/15	14/141/141/14	0.685

The length of hospital stay was calculated for each admission including initial URS and all subsequent auxiliary procedures. There was no statistical significance in the length of hospital stay and the total number of admissions to reach a stone-free status between the groups (Table 5).

**Table 5.** Length of stay and costing.

Variable	Group 1 Laser lithotripsy(*N* = 48)	Group 2 Pneumatic lithotripsy(*N* = 53)	*P*
Hospital stay for URS, days, mean (SD)	1.1 (0.308)	1.11 (0.375)	0.896
No. of admissions, median (range)	1 (1–3)	1 (1–4)	
Total hospital stay, mean (SD)	1.261 (0.48)	1.339 (0.764)	0.54
Costs for URS, Egyptian Pounds, mean (SD)	6928.12 (347.77)	3251.13 (272.79)	<0.001*
Cost to attain stone-free status, Egyptian Pounds, mean (SD)	7286.17 (689.49)	3404.11 (497. 26)	<0.001*

*Values statistically significant at *P* < 0.05.

The use of the laser was associated with >200% increase in costs, with no significant decrease in hospital stay, auxiliary procedures, or the number of admissions to reach a stone-free status, as well as complications (Table 5).

## Discussion

The management of stone disease has been revolutionised. The miniaturisation of scopes and sophistication of medical instrumentation is driving urological practice into a high-technological performance with its economic impact, particularly in developing countries [2,13].

URS with lithotripsy is the benchmark treatment for large mid-ureteric stones. Despite the availability of several energy sources, pneumatic and laser energy are favoured for their high SFRs (>90%) [8] and lower morbidity rates [13,14].

Recently, there has been a trend favouring the use of laser energy to treat ureteric stones. This shift may be driven by the laser’s high SFR, capability to fragment all types of stones, lower incidence of stone migration [15], multi-purpose laser machines installed in medical facilities, and even the marketing effect of lasers influencing requests by patients. In the absence of objective benefits both for the patient and the community, this represents a huge financial burden.

In our present study, we attempted to evaluate the results of both pneumatic and holmium laser lithotripsy in the management of large (>10 mm) mid-ureteric stones in a tertiary referral hospital in a developing country. There were no statistically significant differences in demographic data regarding stone size, laterality, and number in both groups.

Operative time was significantly longer when pneumatic lithotripsy was used, which is discordant with most published literature [16,17]. We believe this difference is related to the combined fragmentation/dusting technique used with the laser, this results in smaller fragments, thus reducing the need for fragment retrieval in comparison to pneumatic lithotripsy in which additional time is needed for retrieval of relatively larger stone fragments.

Due to the small laser fibre size compared with the pneumatic probe, better irrigation and consequently vision can be achieved.

In the present study, at the end of the procedure the ureter was inspected and retrograde pyelography was done, if there were no mucosal tears or extravasation of the dye, the insertion of a ureteric stent was omitted. Stents were used in >80% of the cases, with no difference in the rate of stenting between both groups. External stenting using a ureteric catheter was used more frequently than the internal JJ stent in both groups.

An internal stent was the choice in cases of ureteric injuries, when there was the possibility of residual fragments, and surgeon preference. The use of internal stents did not affect the length of hospital stay nor complications, although it added an auxiliary procedure especially in absence of residual fragments. We believe that in the absence of significant stone fragments, gross haematuria, or a ureteric injury beyond a mucosal tear documented by retrograde pyelography at the end of the procedure, that the use of an external stent is safe and cost-effective [18].

Upward migration of a ureteric stone is a common complication when using a pneumatic energy source [19] and is one of the arguments favouring the use of laser lithotripsy. In our present study, no difference was found in the rate of stone migration between the groups. Stone migrations occurred in four patients (two in each group). Three of the patients had more than one stone, and the stone size was >15 mm. Patient numbers were insufficient for subgroup analysis, so a correlation between the incidence of stone migration in relation to stone burden and number needs to be further evaluated. The low rate of stone migration using pneumatic lithotripsy in our present study may be explained by the fact that URS for proximal stones is usually done by an experienced urologist at our centre.

The published URS complication rate varies between 9% and 25% [20,21], with the Ureteroscopy Global Study [21] reporting that most of them are minor. The Clavien–Dindo Grade III, IV and V complication rates were 0.5%, 0.1%, and 0.02%, respectively. In our present study, there was no statically significant difference in complications between the groups. All the complications were Clavien–Dindo Grade <III and were managed conservatively. The type of lithotripsy used did not affect the complication rate. In their study, Bapat et al. [22] and Tipu et al. [23] reported a significantly lower complication rate in patients undergoing laser lithotripsy, whereas Kassem et al. [20] and Bora et al. [14] reported no significant difference between the two groups in terms of complications.

In the present study, the SFRs were evaluated on postoperative day 1 and day 30, using a combined KUB and US protocol [12]. The SFRs were 93% and 92% on Day 1 and reached 98% and 96% on Day 30 in the laser and pneumatic groups, respectively, with no statistically significant differences. A previous comparison of SFR using laser vs pneumatic lithotripsy was in favour of the laser group [17]; however, a large prospective randomised study in 2015 concluded that SFRs were equal for laser and pneumatic lithotripsy at 3 months [8].

The financial burden of using laser lithotripsy was questioned in our present study. The use of laser in comparison to pneumatic lithotripsy did not affect the number or the type of auxiliary procedures. The mean (SD) hospital stay was 1.261 (0.48) days and 1.339 (0.764) days in the laser and pneumatic lithotripsy groups, respectively. Calculation of the direct costs related to the procedures, disposable supplies, and hospital stay, in addition to the maintenance and depreciation costs of the laser and pneumatic machines, showed a significant economic benefit favouring the use of pneumatic lithotripsy. This is the first study in a developing country to address the economic aspects of this medical service.

We acknowledge that our present study has some limitations. A larger number of patients are needed to confirm the results. Patients with previous stone treatment were not included in our present study. The study design did not include stone density and we used high-power laser machines, which may have higher running costs in comparison to lower energy ones. Furthermore, the relationship between stone burden and stone migration was not analysed. The economic aspect discussed evaluated only the costs of service provided without taking into consideration the benefits and costs needed for a physician to learn and acquire new techniques and technologies.

## Conclusion

URS with lithotripsy is considered the standard treatment for large mid-ureteric stones; our present study showed that the type of lithotripsy did not affect the SFR or complications. A considerable cost increase is seen when laser lithotripsy is used, which puts a huge financial burden on health service providers, especially in underdeveloped and developing economies.

## References

[1] Segura JW, Preminger GM, Assimos DG, et al. Ureteral stones clinical guidelines panel summary report on the management of ureteral calculi. The American urological association. J Urol. 1997;158:1915–1921. 9334635 10.1016/s0022-5347(01)64173-9

[2] Geraghty RM, Jones P, Somani BK. Worldwide trends of urinary stone disease treatment over the last two decades: a systematic review. J Endourol. 2017;31:547–556. 28095709 10.1089/end.2016.0895

[3] Doizi S, Raynal G, Traxer O. Evolution of urolithiasis treatment over 30 years in a French academic institution. Prog Urol. 2015;25:543–548. 26094095 10.1016/j.purol.2015.05.002

[4] Zumstein V, Betschart P, Abt D, et al. Surgical management of urolithiasis - a systematic analysis of available guidelines. BMC Urol. 2018;18:25. 29636048 10.1186/s12894-018-0332-9 PMC5894235

[5] Parker BD, Frederick RW, Reilly TP, et al. Efficiency and cost of treating proximal ureteral stones: shock wave lithotripsy versus ureteroscopy plus holmium: yttrium-aluminum-garnetlaser. Urology. 2004;64:1102–1106. 15596177 10.1016/j.urology.2004.07.040

[6] Kadyan B, Sabale V, Mane D, et al. Large proximal ureteral stones: ideal treatment modality? Urol Ann. 2016;8:189–192. 27141190 10.4103/0974-7796.157963 PMC4839237

[7] Pietropaolo A, Proietti S, Geraghty R, et al. Trends of ‘urolithiasis: interventions, simulation, and laser technology’ over the last 16 years (2000-2015) as published in the literature (PubMed): a systematic review from European section of Uro-technology (ESUT). World J Urol. 2017;35:1651–1658. 28593477 10.1007/s00345-017-2055-z PMC5649597

[8] Li L, Pan Y, Weng Z, et al. A prospective randomized trial comparing pneumatic lithotripsy and holmium laser for management of middle and distal ureteral calculi. J Endourol. 2015;29:883–887. 25578351 10.1089/end.2014.0856

[9] Sharif AI. The rocky road to universal health coverage in Egypt: A political economy of health insurance reform from 2005–2015. Int Soc Secur Rev. 2018;71:79–101.

[10] Chen S, Zhou L, Wei T, et al. Comparison of holmium: YAG laser and pneumatic lithotripsy in the treatment of ureteral stones: an update meta-analysis. Urol Int. 2017;98:125–133. 27505176 10.1159/000448692

[11] Dindo D, Demartines N, Clavien PA. Classification of surgical complications: a new proposal with evaluation in a cohort of 6336 patients and results of a survey. Ann Surg. 2004;240:205–213. 15273542 10.1097/01.sla.0000133083.54934.ae PMC1360123

[12] Ahmed AF, Gabr AH, Emara AA, et al. Factors predicting the spontaneous passage of a ureteric calculus of ≤10 mm. Arab J Urol. 2015;13:84–90. 26413326 10.1016/j.aju.2014.11.004 PMC4561928

[13] Sancak EB, Kılınç MF, Yücebaş SC. Evaluation with decision trees of efficacy and safety of semirigid ureteroscopy in the treatment of proximal ureteral calculi. Urol Int. 2017;99:320–325. 28472804 10.1159/000474954

[14] Bora I, Volkan S, Oğuzcan E, et al. Comparison of efficacy and complications of holmium laser and pneumatic lithotripters used in the ureterorenoscopic treatment of proximal ureter stones, a multi-center study of society of urological surgery aegean study group. J Urol Surg. 2018;5:158–163. .

[15] Kronenberg P, Somani B. Advances in lasers for the treatment of stones – a systematic review. Curr Urol Rep. 2018;19:45. 29774438 10.1007/s11934-018-0807-y PMC5958148

[16] Khoder WY, Bader M, Sroka R, et al. Efficacy and safety of Ho: yAGlaser lithotripsy for ureteroscopic removal of proximal and distal ureteral calculi. BMC Urol. 2014;14:62. 25107528 10.1186/1471-2490-14-62 PMC4132277

[17] Abedi AR, Razzaghi MR, Allameh F, et al. Pneumatic lithotripsy versus laser lithotripsy for ureteral stones. J Lasers Med Sci. 2018;9:233–236. 31119016 10.15171/jlms.2018.42 PMC6499559

[18] Mittakanti HR, Conti SL, Pao AC, et al. Unplanned emergency department visits and hospital admissions following ureteroscopy: do ureteral stents make a difference? Urology. 2018;117:44–49. 29601836 10.1016/j.urology.2018.03.019

[19] Sen H, Bayrak O, Erturhan S, et al. Comparing of different methods for prevention stone migration during ureteroscopic lithotripsy. Urol Int. 2014;92:334–338. 23838044 10.1159/000351002

[20] Kassem A, Elfayoumy H, Elsaied W, et al. Laser and pneumatic lithotripsy in the endoscopic management of large ureteric stones: a comparative study. Urol Int. 2012;88:311–315. 22441150 10.1159/000336254

[21] De la Rosette J, Denstedt J, Geavlete P, et al. The clinical research office of the endourological society ureteroscopy global study: indications, complications, and outcomes in 11,885 patients. J Endourol. 2014;28:131–139. 24147820 10.1089/end.2013.0436

[22] Bapat SS, Pai KV, Purnapatre SS, et al. Comparison of holmium laser and pneumatic lithotripsy in managing upper-ureteral stones. J Endourol. 2007;21:1425–1427. 18186678 10.1089/end.2006.0350

[23] Tipu SA, Malik HA, Mohhayuddin N, et al. Treatment of ureteric calculi–use of holmium: yAGlaser lithotripsy versus pneumatic lithoclast. J Pak Med Assoc. 2007;57:440–443. 18072637

